# Impact of Co-Mutations and Genetic Variations on Malignancy Risk in *RAS*-Positive Indeterminate Thyroid Nodules: an Institutional Experience

**DOI:** 10.1007/s12022-026-09910-6

**Published:** 2026-03-05

**Authors:** Lawrence Q. Wong, Zubair W. Baloch

**Affiliations:** Department of Pathology and Laboratory Medicine, Perelman School of Medicine, University of Pennsylvania, 6 Founders Pavilion, 3400 Spruce Street, Philadelphia, PA 19104 USA

**Keywords:** Thyroid nodule, *RAS* mutation, *NRAS*/*HRAS*/*KRAS*, Indeterminate cytology, Follicular-patterned lesions, NIFTP, IEFVPTC, Next-generation sequencing

## Abstract

Mutations in *RAS* proto-oncogenes (*NRAS*, *HRAS*, *KRAS*) are common in thyroid nodules, though their prognostic significance remains unclear. This retrospective study analyzed 354 thyroid nodules from 346 patients (2018–2023) to investigate the clinical and pathological implications of isolated *RAS* mutations and *RAS* with co-occurring genetic alterations. Isolated *RAS* mutations were found in 41.0% (*n* = 145), while 54.8% (*n* = 194) had *RAS* with additional molecular alterations; *NRAS* was the most frequent subtype (62.1%). Among co-occurring mutations, *EIF1AX* (46.7%) and *TERT* (26.7%) were the most common, primarily in *NRAS*-positive cases. Surgical follow-up data from 302 cases revealed a malignancy rate of 52.3% (*n* = 158), with 60.1% (*n* = 95) being invasive encapsulated follicular variant of papillary thyroid carcinoma (IEFVPTC). *NRAS* mutations appeared in 64.6% of malignant cases. Isolated *RAS* mutations were mainly associated with benign/low-risk neoplasms (47.0%), notably follicular adenomas and encapsulated non-invasive follicular thyroid neoplasm with papillary-like nuclear features (NIFTP), or malignancies (41.0%). The malignancy rate was higher in nodules with a *RAS* mutation plus one concomitant molecular alteration (54.3%), and nearly 100% in those with three additional genetic alterations. Co-occurring genetic alterations with *RAS* mutations markedly increased the risk of malignancy compared with isolated *RAS* mutations (Fisher’s exact test, two-tailed *p* = 0.0026) and were associated with more aggressive tumor phenotypes, whereas isolated *RAS* mutations were more common in indolent neoplasms. Comprehensive molecular profiling is essential for accurate risk stratification and management of indeterminate thyroid nodules.

## Introduction

Thyroid cancer is the most common endocrine malignancy, with about 44,020 new cases annually in the U.S. alone [1]. Fine-needle aspiration (FNA) biopsy is essential for evaluating thyroid nodules, offering a minimally invasive method to assess malignancy risk. The Bethesda System for Reporting Thyroid Cytopathology (TBSRTC) was developed to standardize cytologic interpretation, ensuring consistent and clear communication across institutions [2]. While most nodules are clearly benign or malignant, some fall into indeterminate categories (e.g., atypia of undetermined significance (AUS), follicular neoplasm (FN), or suspicious for malignancy (SM)), where the cytologic results are inconclusive [3, 4]. These cases often lead to unnecessary surgeries, increased patient anxiety, and delayed treatment. To address this, next-generation sequencing (NGS) and molecular analysis are increasingly used to differentiate benign from malignant nodules [5]. ThyroSeq^®^ Genomic Classifier (GC) uses NGS to analyze 112 thyroid-related genes for mutations, fusions, copy number alterations, and gene expression changes linked to thyroid cancer [6]. ThyroSeq^®^ provides a personalized report that categorizes the nodule as either positive or negative for malignancy, detailing any genetic alterations and estimating malignancy risk. This advanced molecular profiling improves risk stratification in indeterminate cases and can help guide clinical decisions, potentially reducing unnecessary surgeries.

The *RAS* family mutations, including *HRAS*,* NRAS*, and *KRAS*, are among the most frequently observed genetic alterations in indeterminate thyroid nodules. Notably, these mutations can be found in both benign and malignant lesions upon surgical resection, underscoring their diagnostic ambiguity [7, 8]. The aim of this study is to retrospectively analyze thyroid FNA cases that tested positive for *RAS* mutations using the ThyroSeq^®^ Version 3 GC between 2018 and 2023. Data were collected on patient demographics, nodule characteristics, cytologic diagnoses based on TBSRTC, and corresponding surgical pathology outcomes. The primary objective is to evaluate the correlation between *RAS* mutation status and both cytologic interpretation and the final histopathologic diagnoses. By systematically reviewing these parameters, this study seeks to clarify the clinical significance of *RAS* mutations in indeterminate thyroid nodules, ultimately aiming to improve diagnostic accuracy, guide appropriate clinical management, and enhance prognostic assessment for affected patients.

## Materials and methods

The study was approved by the University of Pennsylvania Health System Institutional Review Board. A retrospective search of our laboratory information system was performed to identify thyroid FNA cases with any *RAS* mutation identified on subsequent ThyroSeq^®^ V3 GC testing from 2018 to 2023 and relevant cytologic and/or surgical follow-up information. Follow-up in this study refers to the availability of cytology or surgical pathology at the time of review and does not represent longitudinal clinical surveillance. All thyroid FNAs are reviewed by a staff cytopathologist using criteria defined by TBSRTC. Data points collected for this study included: patient demographics, size of the thyroid nodule sampled, aspirator, pathologist rendering the final diagnosis, cytologic diagnosis, ThyroSeq^®^ GC classification, and applicable cytologic and/or surgical follow-up. Relevant surgical pathology follow-up was matched to the size and location of the index nodule. Histopathologic diagnoses were assigned following the nomenclature and criteria recently revised in the 2022 WHO Classification of Thyroid Neoplasms [9].

For the purposes of this study, surgically resected lesions were categorized as thyroid follicular nodular disease (TFND), benign/low-risk neoplasms, or malignant neoplasms in accordance with the WHO 5th edition classification. Lesions previously classified as hyperplastic, adenomatoid nodules, or as multinodular colloid goiter, were reclassified as TFND. Malignancy status was determined based on the final histopathologic diagnosis and served as the reference standard. The term “not otherwise specified (NOS)” was used when a gene mutation was reported but detailed molecular data were incomplete or unavailable. Copy number alterations (CNAs) classified as *RAS*-like were treated as primary *RAS* alterations. Cases with a *RAS*-like CNA and complete molecular profiling showing no additional alterations were classified as isolated *RAS* alterations. Data processing was performed on Microsoft Excel software (Microsoft, Redmond, WA, USA). Fisher’s exact test was used to compare outcomes between cases with isolated *RAS* mutations (no additional genetic alterations detected) and cases with *RAS* mutations plus one or more co-alterations. Second opinion cases with unavailable molecular profiling data (*RAS*-ND, not determined) were excluded from this analysis.

A total of 354 thyroid FNA cases from 346 patients met the inclusion criteria for this study, including 259 in-house cases and 95 second opinion cases. Surgical pathology follow-up was available for 302 cases and served as the reference standard for malignancy determination. All in-house FNAs were performed by a team of endocrinologists or radiologists using 25–27-gauge needles under ultrasound guidance, typically with 2–3 passes per nodule. Each pass yielded an air-dried slide, an alcohol-fixed slide, and residual material rinsed into PreservCyt^®^ solution (Hologic, Marlborough, MA, USA) for ThinPrep^®^ preparation. Rapid on-site evaluation (ROSE) was conducted in nearly all in-house cases by either a cytopathologist or a cytologist. Based on ROSE findings and clinical/radiologic impressions, an additional pass was collected for potential ThyroSeq^®^ GC testing. For second opinion cases, slides were re-reviewed by an in-house cytopathologist, and included confirmed *RAS* mutations by ThyroSeq^®^ GC.

## Results

This study includes 354 thyroid nodules with *RAS* mutations from 346 patients (267 females, 79 males), with a mean age of 51 ± 16 years for females and 53 ± 15 years for males (Table 1). Nodule location was right lobe in 191 cases, left lobe in 148, and isthmus in 15. The mean size was 2.8 cm: 2% were < 1.0 cm, 33.3% between 1.1 and 2.0 cm, 27.4% between 2.1 and 3.0 cm, 17.2% between 3.1 and 4.0 cm, 14.7% >4.1 cm, and 5.4% unspecified.

**Table 1:** Demographic characteristics of *RAS*-positive cytologic thyroid nodules

Variable		Value
Patients, n		346
Female		267
Male		79
Thyroid Nodules, n		354
Right		191
Left		148
Isthmus		15
Age, mean ± SD, y		51 ± 16
Size, cm	n (%)	Mean ± SD, cm
< 1.0	7 (2.0)	0.9 ± 0.14
1.1–2.0	118 (33.3)	1.6 ± 0.26
2.1–3.0	97 (27.4)	2.5 ± 0.28
3.1–4.0	61 (17.2)	3.5 ± 0.28
> 4.1	52 (14.7)	5.3 ± 1.0
NOS	19 (5.4)	
Size, mean ± SD, cm (excluding NOS)		2.8 ± 1.4

NOS: Not otherwise specified

Cytologic diagnoses (TBSRTC) were Bethesda III (AUS) in 198 cases (55.9%), Bethesda IV (FN) in 125 (35.3%), Bethesda V (SM) in 29 (8.2%), and Bethesda II in 2 (0.6%). The two Bethesda II cases were second opinion cases reclassified as such upon review. ROSE was performed in 197 cases (55.6%); preliminary interpretations included AUS (61.9%), adequate (15.2%), FN (14.2%), neoplastic (5.0%), benign follicular nodule (3.0%), and suspicious for papillary thyroid carcinoma (0.5%).

Table 2 summarizes *RAS*-positive thyroid nodules and additional molecular alterations. *NRAS* mutations were most frequent (*n* = 220, 62.1%), followed by *HRAS* (*n* = 81, 22.9%) and *KRAS* (*n* = 48, 13.6%); 1 case had confirmed *RAS* mutation but the isoform was unknown and classified as *RAS*-NOS, four cases had molecular events that mimic *RAS* signaling and was classified as *RAS*-like. Overall, 41.0% (*n* = 145) had isolated *RAS* mutations, while 54.8% (*n* = 194) had *RAS* plus additional molecular alterations, including abnormal gene expression profiles (*n* = 146, 41.2%), copy number changes (*n* = 62, 17.5%), and/or other mutations (*n* = 45, 12.7%). Specifically, 40.4% (*n* = 143) had *RAS* plus one additional genomic alteration (*RAS* + 1), 12.7% (*n* = 45) had two (*RAS* + 2), and 1.7% (*n* = 6) had three (*RAS* + 3).

**Table 2:** Degree of co-occurring molecular alterations in *RAS*-positive nodule

*RAS* Mutation	*RAS* only	*RAS* + 1	*RAS* + 2	*RAS* + 3	*RAS*-ND	*RAS* + Other Mutation	*RAS* + Copy Number Alterations	*RAS* + Abnormal Gene Expression Profile
*HRAS*	28	37	13	0	3	5	16	41
*KRAS*	21	22	3	0	2	6	1	20
*NRAS*	94	81	29	6	10	32	44	81
*RAS*-NOS	0	1	0	0	0	1	0	0
*RAS*-like	2	2	0	0	0	1	1	4

*RAS*-NOS (not otherwise specified): *RAS* mutation, isoform unknown; *RAS*-like: molecular events that mimic *RAS* signaling; *RAS* + 0: *RAS* mutation with no other molecular alterations detected, *RAS* + 1: *RAS* mutation with 1 other molecular alteration detected, *RAS* + 2: *RAS* mutation with 2 other molecular alterations detected, *RAS* + 3: *RAS* mutation with 3 other molecular alterations detected, *RAS*-ND (not determined): *RAS* mutation but unavailable data on other molecular alterations

Four *HRAS* variants were identified, with p.Q61R (c.182 A > G) being the most frequent (*n* = 48, 59.3%), followed by p.Q61K (c.181 C > A) (*n* = 15), p.G13R (c.37G > C) (*n* = 10), and p.G13V (c.38G > T) (*n* = 1). Seven cases were *HRAS* NOS, and 61.7% (*n* = 50) had additional molecular alterations. Ten *KRAS* variants were identified, with p.Q61R (c.182 A > G) (*n* = 12) and p.G12D (c.35G > A) (*n* = 11) as the most common, followed by p.G12V (c.35G > T) (*n* = 8) and p.G13D (c.38G > A) (*n* = 4). Less frequent variants included p.G12R (c.34G > C) (*n* = 2), p.G13R (c.37G > C) (*n* = 1), p.G13E (c.38_39delinsAA) (*n* = 1), p.Q61K (c.180_181delinsAA) (*n* = 1), p.A146T (c.436G > A) (*n* = 1), and p.A146V (c.437 C > T) (*n* = 1). Six cases were classified as *KRAS* NOS. Over half (*n* = 25, 52.1%) had additional molecular alterations. Five *NRAS* variants were detected, with p.Q61R (c.182 A > G) being predominant (*n* = 147, 66.8%), followed by p.Q61K (c.181 C > A) (*n* = 34), p.G13R (c.37G > C) (*n* = 8), p.G12D (c.35G > A) (*n* = 1), and p.Q61L (c.182 A > T) (*n* = 1). Twenty-nine cases were *NRAS*-NOS, and 52.7% (*n* = 116) had additional genetic alterations. Of the second opinion cases, 28 (34.6%) were *HRAS*, 15 (31.3%) were *KRAS*, 50 (22.8%) were *NRAS*, and 2 (50.0%) had *RAS*-like genetic alterations.

A total of 45 *RAS*-positive nodules harbored concomitant mutations, all but two were surgically resected. *EIF1AX* was the most frequent co-mutation (*n* = 21, 46.7%), followed by *TERT* (*n* = 13, 28.9%). Most additional mutations (*n* = 32, 71.1%) occurred in *NRAS*-positive nodules. The most common combinations were *NRAS + EIF1AX* (*n* = 18, 40.0%), *NRAS + TERT* (*n* = 6, 13.3%), *KRAS + TERT* (*n* = 4, 8.9%), and *NRAS + TP53* (*n* = 4, 8.9%).

Co-occurring variants included alterations in *DICER1* (p.D1709E (c.5127T > A)), *EIF1AX* (p.G9R (c.25G > C), p.G9V (c.26G > T), splice-site variant, variant not otherwise specified), *EZH1* (p.P573S (c.1717 C > T), p.Q571R (c.1712 A > G)), *NRAS* (p.Q61K (c.181 C > A)), *PTEN* (p.I101S (c.302T > G), p.R173Hfs7 (c.518_522delinsACTATG)), *TERT* (c.–124 C > T [C228T], c.–146 C > T [C250T]), *TP53* (p.C275S (c.824G > C), p.E171G (c.512 A > G), p.G244V (c.731G > T), p.R280 (c.838 A > T), p.V272L (c.814G > T)), and *TSHR* (p.S281N (c.842G > A), p.D619V (c.1856 A > T)).

Histopathology follow-up data were available for 302 (85.3%) cases, 1 (0.3%) had a repeat FNA, and 51 (14.4%) had no documented follow-up (Table 3). The median time to surgical excision was 52 days from the initial FNA. Procedures included lobectomy in 204 cases (67.3%), total thyroidectomy in 96 (31.7%), isthmusectomy in 2 (0.7%), and repeat FNA in 1 (0.3%). Average nodule size was 2.45 cm for lobectomy, 3.10 cm for total thyroidectomy, 2.05 cm for isthmusectomy, and 1.6 cm for repeat FNA.

**Table 3:** Follow-up characteristics of *RAS*-positive cytologic nodules

	Value
Follow-up Type, *n*	
No Follow-up	51
Surgical Follow-up	302
Cytology Follow-up	1
Elapsed, median, d	52
Procedure, n	303
Lobectomy/Hemithyroidectomy	204
Total Thyroidectomy	96
Isthmusectomy	2
Repeat FNA	1

Overall, 52.3% (*n* = 158) of follow-up cases were malignant, 60.1% (*n* = 95) were invasive encapsulated follicular variant of papillary thyroid carcinoma (IEFVPTC). Follicular thyroid carcinoma (FTC) comprised 9.5% (*n* = 15), while classical and other subtypes of papillary thyroid carcinoma (PTC) accounted for 20.9% (*n*= 33). Benign/low-risk neoplasms accounted for 40.7% (*n* = 123) of cases, including 53.7% (*n* = 66) encapsulated non-invasive follicular thyroid neoplasm with papillary-like nuclear features (NIFTP), 37.4% (*n* = 46) follicular adenomas (FA), and 8.9% (*n* = 11) oncocytic adenomas (OA).

For surgically resected cases, cytology-histopathology correlation was as follows: among Bethesda III nodules, 9.0% (*n* = 15) were classified as TFND, 47.0% (*n* = 78) benign/low-risk neoplasms, and 44.0% (*n* = 73) malignant (Table 4), with a malignancy rate of 44.0%. For Bethesda IV nodules, 5.4% (*n* = 6) were TFND, 33.3% (*n* = 37) benign/low-risk neoplasms, and 61.3% (*n* = 68) malignant; the malignancy rate was 61.3%. Bethesda V nodules had no TFND on excision; 32.0% (*n* = 8) were benign/low-risk neoplasms and 68.0% (*n* = 17) malignant, the malignancy rate was 68.0%. Follicular adenoma (*n* = 46) and NIFTP (*n* = 66) comprised 91.1% of all benign/low-risk neoplasm diagnoses. When NIFTP cases were considered malignant, the malignancy rate increased to 68.1% for Bethesda III, 78.4% for Bethesda IV, and 96.0% for Bethesda V. Among malignant nodules, IEFVPTC represented 60.1% (*n* = 95) of cases.

**Table 4:** Cytology, follow-up diagnosis, and *RAS* mutation type in *RAS*-positive nodules

	CP: Indeterminate, *n*	SP: TFND, *n*	SP: Benign/Low-Risk Neoplasm, *n*	SP: Malignant, *n*
TBSRTC				
Bethesda II	1	0	0	0
Bethesda III	0	15	78	73
Bethesda IV	0	6	37	68
Bethesda V	0	0	8	17
Total	1	21	123	158
*RAS* Mutation				
* HRAS*	0	4	32	38
* KRAS*	1	0	17	18
* NRAS*	0	17	70	102
* RAS*-NOS	0	0	1	0
* RAS*-like	0	0	3	0

TBSRTC: The Bethesda System for Reporting Thyroid Cytopathology; CP: Cytopathology; SP: Surgical Pathology; SP: TFND (thyroid follicular nodular disease) – includes multinodular goiter, hyperplastic nodule, adenomatous nodule, or adenomatous hyperplasia; SP: Benign/low-risk neoplasms – includes: FA (follicular adenoma), OA (oncocytic adenoma), and NIFTP (non-invasive follicular thyroid neoplasm with papillary-like nuclear features); SP: Malignant neoplasms – includes: FTC (follicular thyroid carcinoma), IEFVPTC (invasive encapsulated follicular variant of papillary thyroid carcinoma), PTC (papillary thyroid carcinoma), OCA (oncocytic carcinoma), PDTC (poorly differentiated thyroid carcinoma), MTC: medullary thyroid carcinoma; *RAS*-NOS (not otherwise specified): *RAS* mutation, isoform unknown; *RAS*-like: molecular events that mimic *RAS* signaling

**Fig. 1:**
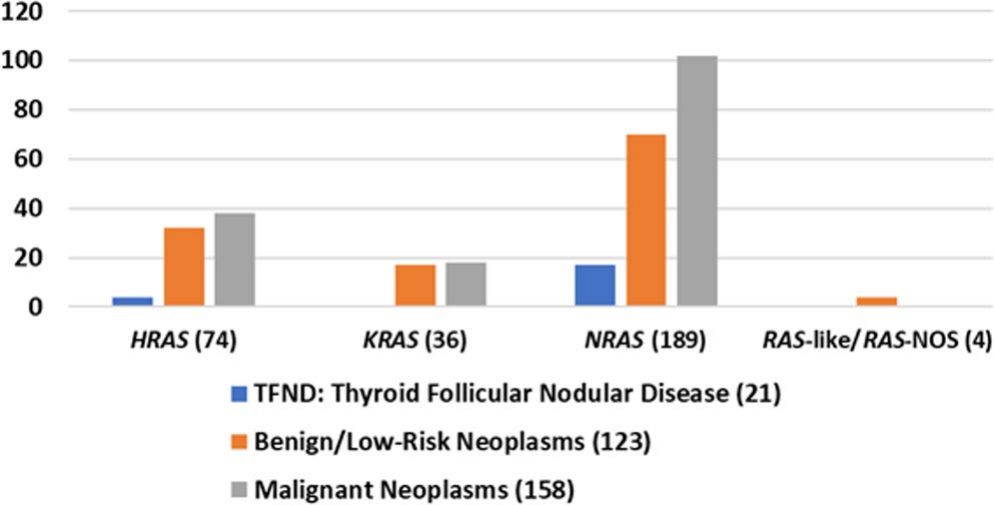
Distribution of *HRAS*, *KRAS*, *NRAS*, or *RAS*-like/*RAS*-NOS mutation among TFND, benign/low-risk neoplasm, and malignant surgically resected nodules. TFND (Thyroid follicular nodular disease) – includes multinodular goiter, hyperplastic nodule, adenomatous nodule, or adenomatous hyperplasia; Benign/low-risk neoplasms – includes: FA (follicular adenoma), OA (oncocytic adenoma), and NIFTP (non-invasive follicular thyroid neoplasm with papillary-like nuclear features); Malignant neoplasms – includes: FTC (follicular thyroid carcinoma), IEFVPTC (invasive encapsulated follicular variant of papillary thyroid carcinoma), PTC (papillary thyroid carcinoma), OCA (oncocytic carcinoma), PDTC (poorly differentiated thyroid carcinoma), and MTC: medullary thyroid carcinoma; NOS: Not otherwise specified

Histopathologic follow-up, along with the molecular profile, is illustrated in Fig. 1 and is summarized in Table 4. Among follow-up cases, *HRAS* mutations were found in 74 (24.5%), *KRAS* in 35 (11.6%), and *NRAS* in 189 (62.6%). In surgically resected cases, malignant neoplasms were found across all mutation types: *HRAS* (*n* = 38, 51.4%), *KRAS* (*n* = 18, 51.4%), and *NRAS* (*n* = 102, 54.0%).

*DICER1* co-mutation was identified in one malignant case. *EIF1AX* co-mutations were identified in 10 benign/low-risk neoplasms and 11 malignant cases. Two *RAS*-positive cytologic nodules harbored co-occurring *EZH1* mutation but did not undergo further follow-up. *PTEN* co-mutations were observed in one benign/low-risk neoplasm and one malignant case. *TERT* promoter co-mutations were found in one benign/low-risk neoplasm case and 12 malignant cases. *TP53* co-mutations were found exclusively in 4 malignant cases; 1 case harbored 2 distinct *TP53* co-mutations. *TSHR* co-mutations were present in one TFND and one benign/low-risk neoplasm case. The risk of malignancy for *RAS* co-mutation with *DICER1* was 100%, 52.4% for *EIF1AX*, 50% for *PTEN*, 92.3% for *TERT*, 100% *TP53*, and 0% *TSHR* mutations (Table 5).

**Table 5:** Co-occurring mutations and diagnostic categories in *RAS*-positive surgically resected thyroid nodules

	*DICER1*	*EIF1AX*	*NRAS*	*PTEN*	*TERT*	*TP53*	*TSHR*
Thyroid follicular nodular disease (TFND)							
TFND	0	0	0	0	0	0	1
Benign/low-risk neoplasms							
FA	0	5	0	1	0	0	0
OA	0	1	0	0	0	0	1
NIFTP	0	4	1	0	1	0	0
Malignant neoplasms							
FTC	0	1	0	1	0	0	0
IEFVPTC	0	8**	0	0	7**	3*	0
PTC	1	2	0	0	2	0	0
OCA	0	0	0	0	2	1	0
PDTC	0	0	0	0	1	1	0
ROM	100%	52.4%	0%	50%	92.3%	100%	0%

TFND (thyroid follicular nodular disease) – includes multinodular goiter, hyperplastic nodule, adenomatous nodule, or adenomatous hyperplasia; Benign/low-risk neoplasms – includes: FA (follicular adenoma), OA (oncocytic adenoma), and NIFTP (non-invasive follicular thyroid neoplasm with papillary-like nuclear features); Malignant neoplasms – includes: FTC (follicular thyroid carcinoma), IEFVPTC (invasive encapsulated follicular variant of papillary thyroid carcinoma), PTC (papillary thyroid carcinoma), OCA (oncocytic carcinoma), and PDTC (poorly differentiated thyroid carcinoma); ROM: Risk of Malignancy

*1 case of IEFVPTC harbored *NRAS* and 2 distinct *TP53* mutations

**1 case of IEFVPTC harbored *NRAS*, *EIF1AX*, and *TERT* mutations

Among the 43 nodules harboring *RAS* mutations with additional molecular alterations, 22 underwent lobectomy and 21 underwent total thyroidectomy (Table 6). Of the total thyroidectomies, 16 cases (76.2%) were malignant and 5 cases (23.8%) were benign/low-risk neoplasms (FA, NIFTP, OA). In comparison, 10 of 22 lobectomy cases (45.5%) were non-malignant and 12 (54.5%) were malignant. For this study, surgical pathology outcomes refer exclusively to the index nodule sampled by FNA; any additional malignant findings outside the index nodule were not considered.

**Table 6:** Outcomes of lobectomy/isthmusectomy and total thyroidectomy in *RAS* + additional mutation nodules

Surgery type	Non-Malignant, *n*	Malignant, *n*	Total, *n*
Lobectomy	10	12	22
Total Thyroidectomy	5	16	21
Total	15	28	43

**Fig. 2:**
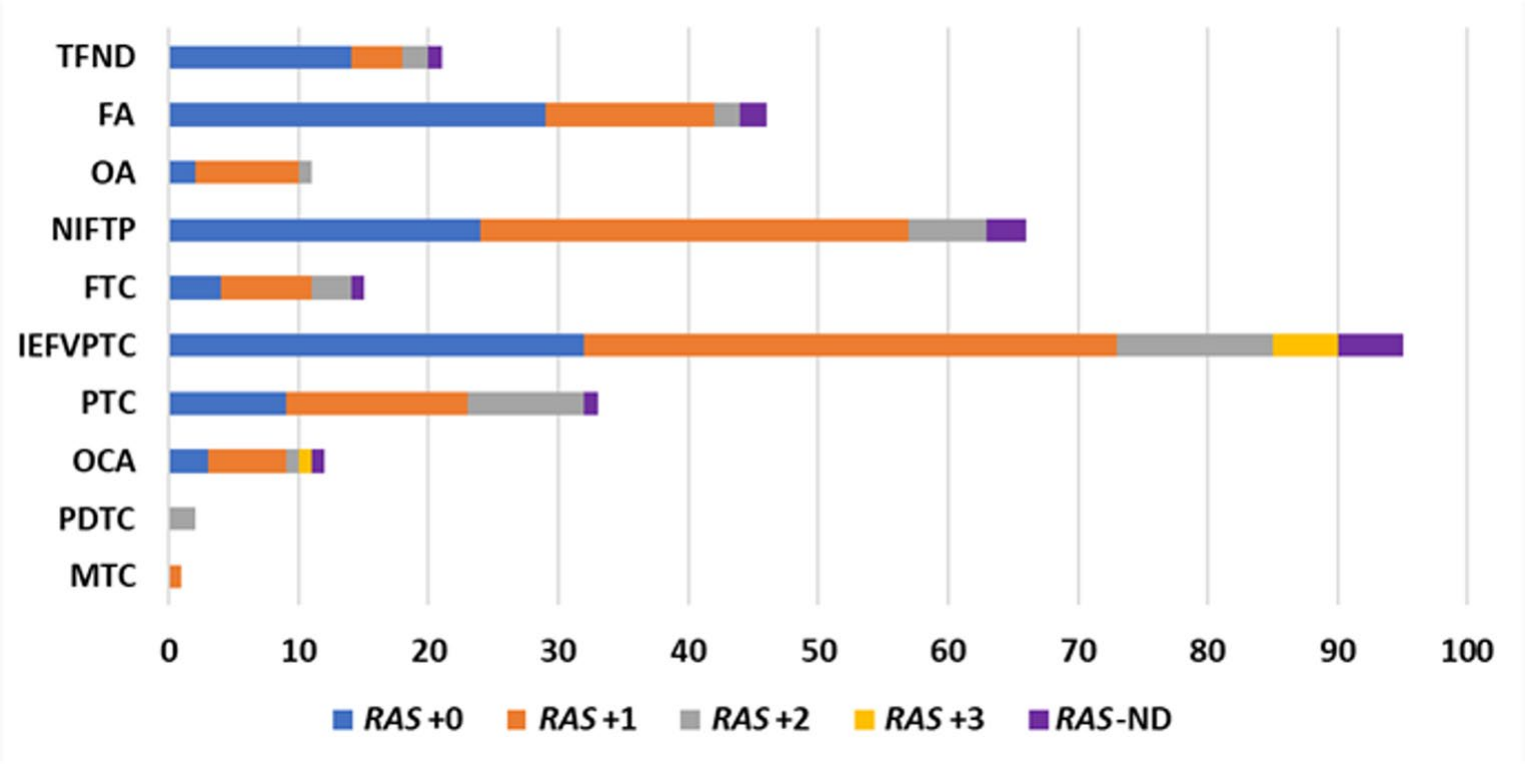
Degree of *RAS* molecular alterations among surgically resected nodules. *RAS*-NOS cases (confirmed *RAS* mutations with unknown isoform) and *RAS*-like alterations were included in the corresponding *RAS* category according to the number of additional alterations. TFND (Thyroid follicular nodular disease) – includes multinodular goiter, hyperplastic nodule, adenomatous nodule, or adenomatous hyperplasia; Benign/low-risk neoplasms - FA: follicular adenoma, OA: oncocytic adenoma, and NIFTP: non-invasive follicular thyroid neoplasm with papillary-like nuclear features (NIFTP); Malignant neoplasms - FTC: follicular thyroid carcinoma, IEFVPTC: invasive encapsulated follicular variant of papillary thyroid carcinoma, PTC: papillary thyroid carcinoma – other subtypes, OCA: oncocytic carcinoma, PDTC: poorly differentiated thyroid carcinoma, MTC: medullary thyroid carcinoma; *RAS* + 0: Isolated *RAS* mutation, *RAS* + 1: *RAS* mutation with 1 other molecular alteration*, *RAS* + 2: *RAS* mutation with 2 other molecular alterations*, *RAS* + 3: *RAS* mutation with 3 other molecular alterations*, *RAS*-ND (not determined): *RAS* mutation but unavailable data on other molecular alterations *Other molecular alterations include an abnormal gene expression profile, copy number alterations, and/or distinct gene mutations in addition to *RAS*

Cases with isolated *RAS* mutations were diagnosed on surgical follow-up as TFND (*n* = 14, 12.0%), benign/low-risk neoplasm (*n* = 55, 47.0%), or malignant (*n* = 48, 41.0%), with follicular adenoma (*n* = 29) more common than NIFTP (*n* = 24), as shown in Fig. 2. The malignancy rate was 41.0%, with the most frequent malignant diagnosis being IEFVPTC (*n* = 32). When a *RAS* mutation was accompanied by one additional molecular alteration (*RAS* + 1), the malignancy rate increased to 54.3% (*n* = 69). Among benign/low-risk neoplasms, NIFTP (*n* = 33) was most common, followed by follicular adenomas (*n* = 13) and oncocytic neoplasms (*n* = 8). IEFVPTC remained the main malignancy (*n* = 41). In cases with two additional genetic abnormalities (*RAS* + 2), 71.1% (*n* = 27) were malignant, with IEFVPTC (*n* = 12) as the most common malignancy, and NIFTP (*n* = 6) for benign/low-risk neoplasms. All *RAS* + 3 cases were malignant (*n* = 6), predominantly IEFVPTC (*n* = 5).

Cases with additional molecular alterations (mutations beyond *RAS*, abnormal gene expression profile, and/or copy number alterations) were compared with cases harboring isolated *RAS* mutations. Statistically significantly higher risk of malignancy was observed in the *RAS* + any additional molecular alterations group (Fisher’s exact test, two-tailed *p* = 0.0026). Fifteen second opinion cases with *RAS* mutations lacked complete molecular profiling (*RAS*-ND) and were excluded from the analysis.

## Discussion

*RAS*, a family of GTP-binding proteins upstream of *BRAF*, regulates cell growth through the MAPK and PI3K-AKT pathways and is mutated in about one-third of human tumors [7]. First identified in thyroid cancer in 1988, *RAS* mutations—particularly in *NRAS*, followed by *HRAS* and *KRAS*—are found in various thyroid tumors, including follicular and papillary types, but are more strongly associated with aggressive forms like poorly differentiated and anaplastic thyroid carcinomas. These mutations, commonly occur at codons 12, 13, and especially 61, alter GTP-binding or GTPase activity, leading to continuous activation of downstream signaling pathways. *NRAS* mutations tend to activate PI3K-AKT, while *KRAS* mutations favor the MAPK pathway. In PTC, *RAS* and *BRAF* mutations are mutually exclusive, indicating *RAS* can independently drive tumor development through aberrant signaling that promotes uncontrolled cell proliferation and differentiation.

*RAS* mutations are commonly found in indeterminate thyroid nodules, with clinical outcomes ranging from indolent to aggressive disease. Several studies have reported mostly benign or slow-growing courses [10–30], while others have described more aggressive clinical courses [31–38]. As molecular testing gains prominence, it has become routine to perform genomic profiling on indeterminate thyroid cytology cases to aid diagnosis and management. Within our healthcare system, all indeterminate cytology specimens undergo reflex testing with ThyroSeq^®^ GC automatically. Following testing, our cytopathologists provide a comprehensive final report with interpretative recommendations tailored to the patient’s molecular profile. This approach enables a more precise understanding of the tumor biology at an individual level, facilitating targeted and personalized clinical interventions [39, 40].

Overall, the presence of a *RAS* mutation is associated with a 1.7-fold increased risk of malignancy, often warranting surgical resection [41]. Across the six-year study period, follow-up data were available for our cohort in 85.6% (*n* = 303) cases, with all but one undergoing surgical resection. Among these, 52.3% (*n* = 158) were malignant, with IEFVPTC comprising 60.1% (*n* = 95) of malignant cases. The malignancy rate increased progressively with higher TBSRTC category, from 44.0% in Bethesda III to 68.0% in Bethesda V nodules. When NIFTP cases were included as malignant on follow-up, the malignancy rates rise substantially to 68.1% for Bethesda III to 96.0% for Bethesda V categories.

A recent meta-analysis by Riccio et al. [41] demonstrated that *RAS* mutations are frequently associated with thyroid tumors with a follicular growth pattern, including IEFVPTC and FTC. Consistent with this, lesions with a follicular growth pattern – including FA, OA, NIFTP, IEFVPTC, and oncocytic carcinoma (OCA) – predominated among *RAS*-positive nodules in our cohort, accounting for 81.1% (245/302). This spectrum highlights the diagnostic challenges posed by *RAS*-mutated lesions, which often overlap morphologically and molecularly between benign and malignant categories [42].

The study by Sfreddo et al. [43] compared outcomes in *RAS*-mutated indeterminate thyroid nodules managed either with active surveillance or immediate surgery. At our institution, most cases underwent prompt surgical resection, with a median interval of 52 days from cytologic diagnosis to surgical pathology. Only five cases had follow-up extending beyond 273 days, reflecting the surgical management approach in our practice.

Consistent with prior studies [41, 44], *NRAS* mutations were the most frequent in our cohort, present in 62.1% (*n* = 220) of cases, followed by *HRAS* in 22.9% (*n* = 81), and *KRAS* in 13.6% (*n* = 48). Overall, *RAS* p.Q61R was the most common hot-spot mutation in our cohort, occurring in 207 of 354 cases (58.5%). Although somewhat lower than the 76% prevalence reported by Alzumaili et al. [45], the predominance of p.Q61R in both cohorts underscores its central role among *RAS*-driven thyroid nodules. The prognostic significance of specific *RAS* subtypes remains an area of ongoing investigation. Radkay et al. [46] suggested that *NRAS* and *KRAS* mutations may be linked to less aggressive disease compared to *HRAS* mutations. However, in our study, malignancy rates were comparable across *RAS* subtypes: 51.4% for *HRAS*, 51.4% for *KRAS*, and 54.0% for *NRAS* indicating similar clinical behavior.

A comprehensive meta-analysis of thirty studies [41] revealed a heterogeneous distribution of *RAS*-positive malignant nodules following surgery: 34% classic subtype of papillary thyroid carcinoma (PTCcs), 39% IEFVPTC, and 23% FTC. In contrast, our cohort showed a predominance of IEFVPTC, which accounted for 60.1% of malignant cases with surgical follow-up. Other malignancies included subtypes of PTC (20.9%), FTC (9.5%), OCA (7.6%), poorly differentiated thyroid carcinoma (1.3%), and medullary carcinoma (0.6%). These findings align with Cameselle-Teijeiro et al. [47] where accurate classification requires integration of morphology with ancillary immunohistochemical and molecular findings.

The study by Bikas et al. [48] demonstrated that patients harboring *RAS* mutations alongside additional genetic alterations tend to exhibit more aggressive disease, with these cumulative genetic “hits” linked to higher mortality risk and greater neoplastic heterogeneity. Reflecting this, our cohort showed a near split between cases with an isolated *RAS* mutation (41.0%) and those with one additional molecular alteration (*RAS* + 1, 40.4%). Notably, the malignancy rate was significantly higher in the *RAS* + 1 group compared to isolated *RAS* cases (54.3% vs. 41.0%), underscoring the impact of coexisting mutations on tumor aggressiveness. When two (*RAS* + 2) or three (*RAS* + 3) additional molecular alterations were present alongside the *RAS* mutation, malignancy rates rose markedly to 71.1% and 100%, respectively. The additional abnormalities identified in our cohort included abnormal gene expression profiles (*n* = 146, 41.2%), copy number alterations (*n* = 62, 17.5%), and other distinct gene mutations beyond *RAS* (*n* = 45, 12.7%).

According to American Thyroid Association (ATA) recommendations, *RAS*-mutated indeterminate nodules may be managed with diagnostic lobectomy or, in select low-risk settings, active surveillance [3]. Our observation that many isolated *RAS* mutation benign/low-risk lesions were follicular adenomas (52.7%) reinforces the appropriateness of conservative or stepwise management strategies consistent with ATA guidance. However, with one or two additional molecular alterations (*RAS* + 1 and *RAS* + 2), the benign-to-low-risk neoplastic population shifted towards NIFTP, comprising 61.1% and 66.7%, respectively. The malignant cohort similarly became more diverse with the addition of genetic alterations; follicular-patterned lesions represented 75.0% of malignancies with isolated *RAS* mutations, 70.0% with *RAS* + 1, and 55.6% with *RAS* + 2. Interestingly, cases with three additional molecular alterations (*RAS* + 3) saw an increase again in follicular-patterned malignancies to 83.3%. These findings highlight how accumulating genetic alterations in *RAS*-mutated thyroid nodules contributes to increasing malignancy risk and complexity of tumor histology, emphasizing the importance of comprehensive molecular profiling for risk stratification and management decisions.

Aside from mutation and genomic variation burden, prior studies highlight that the type of co-occurring alteration with *RAS* strongly influences tumorigenesis and biologic behavior. Isolated *EIF1AX* mutations confer only a modest risk of malignancy (~ 25–40%) [49–51], yet the presence of *EIF1AX* alongside *RAS*, *TERT* promoter, or *TP53* mutations increases the risk to approximately 86%, demonstrating potent cooperative oncogenic effects [51]. Notably, *RAS* + *EIF1AX* co-mutations, especially those involving the A113_splice variant, are consistently enriched in tumors showing progression toward poorly differentiated thyroid carcinoma (PDTC) [52, 53]. In our cohort, the *RAS* + *EIF1AX* combination accounted for 46.7% of all co-mutations with a risk of malignancy of 52.4%, predominantly IEFVPTC; 71.4% involved the A113_splice variant. Aligning with prior observations that *RAS* partnered with *TERT* promoter alterations strongly predicts aggressive clinical disease [54, 55], while *RAS* + *TP53* combinations are similarly associated with dedifferentiation [56, 57]. *RAS* + *TERT* and *RAS* + *TP53* co-mutations were observed in 26.7% and 8.9% of all combinations in our cohort. *RAS* + *TERT* co-mutations had a risk of malignancy of 92.3%, predominantly IEFVPTC with 1 case of PDTC; *RAS* + *TP53* co-mutations were malignant in all cases with 1 case of PDTC. Collectively, these observations illustrate that whereas *RAS* alone often drives indolent or well-differentiated lesions, the acquisition of specific secondary mutations such as *EIF1AX*, *TERT*, or *TP53* can drive tumors towards more aggressive, heterogeneous, and poorly differentiated phenotypes.

Although our data reinforces that additional mutations increase the risk of malignancy in *RAS*-mutated nodules, malignancy is not guaranteed. Notably, 5 patients with *RAS* + additional mutations underwent total thyroidectomy yet were found to have benign/low-risk neoplasms (FA, NIFTP, or OA) on final pathology. This emphasizes the potential for overtreatment and underscores the importance of careful surgical decision-making, particularly when other high-risk clinical or molecular features are absent, consistent with prior reports [58]. At this time, ATA guidelines state that the presence of additional molecular alterations, while informative for risk stratification, should be interpreted alongside cytologic, sonographic, and clinical features rather than used in isolation to determine malignancy or guide definitive surgery [3].

This study has several limitations. Its retrospective design, required for adequate surgical follow-up, limits variable control and may introduce bias. Long-term clinical outcomes were not assessed, so delayed or subsequent malignancies could not be evaluated. Inclusion of both in-house and consultation cases introduced variability in sampling techniques and testing methods. Selection bias is possible due to the exclusive focus on *RAS*-mutant nodules, though this was intentional. Findings may not generalize to nodules with non-diagnostic, benign, or overtly malignant cytology, as most cases had indeterminate cytology (Bethesda III–V). Some cases lacked follow-up, preventing determination of malignancy status—an inherent limitation of retrospective cytology-based studies. Additionally, incomplete ThyroSeq^®^ GC data from outside institutions resulted in classification of some cases as *RAS*-ND and precluded assessment of co-occurring molecular alterations.

Despite these limitations, this study offers several notable strengths. It represents one of the largest single-institution cohorts of *RAS*-mutated thyroid nodules, collected over six years with corresponding surgical follow-up. Approximately one-quarter were second opinion cases, included to enhance diversity and increase sample size, enabling more robust analysis of molecular alterations, histologic subtypes, and clinical outcomes. Importantly, findings were stratified by specific *RAS* point mutations, providing deeper molecular insights. Furthermore, the high rate of surgical resection allowed for strong histopathologic correlation and outcome assessment.

This study underscores that *RAS* mutations, particularly *NRAS*, are common in indeterminate thyroid nodules and frequently co-occur with additional molecular alterations. Our analysis demonstrated that nodules with *RAS* plus additional molecular alterations carry a statistically significantly higher malignancy risk than those with isolated *RAS* mutations. Malignancy risk increases with each added genetic alteration, reaching 100% when three are present. Isolated *RAS* mutations are more often associated with benign-to-low-risk neoplasms such as follicular adenoma or NIFTP, whereas co-mutations correlate with invasive malignancy, especially IEFVPTC. These findings highlight the critical role of comprehensive molecular profiling in refining risk stratification and guiding surgical decision-making for *RAS*-positive nodules.

## Data Availability

No datasets were generated or analysed during the current study.
